# The influence of transketolase on lipid biosynthesis in the yeast *Yarrowia lipolytica*

**DOI:** 10.1186/s12934-020-01398-x

**Published:** 2020-07-11

**Authors:** Adam Dobrowolski, Aleksandra M. Mirończuk

**Affiliations:** Department of Biotechnology and Food Microbiology, Wroclaw University of Environmental and Life Sciences, Chełmońskiego 37, 51-630 Wrocław, Poland

**Keywords:** Pentose phosphate pathway (PPP), Lipids, *Yarrowia lipolytica*, Transketolase

## Abstract

**Background:**

During the pentose phosphate pathway (PPP), two important components, NADPH and pentoses, are provided to the cell. Previously it was shown that this metabolic pathway is a source of reducing agent for lipid synthesis from glucose in the yeast *Yarrowia lipolytica*. *Y. lipolytica* is an attractive microbial host since it is able to convert untypical feedstocks, such as glycerol, into oils, which subsequently can be transesterified to biodiesel. However, the lipogenesis process is a complex phenomenon, and it still remains unknown which genes from the PPP are involved in lipid synthesis.

**Results:**

To address this problem we overexpressed five genes from this metabolic pathway: transaldolase (TAL1, *YALI0F15587*g), transketolase (TKL1, *YALI0E06479*g), ribulose-phosphate 3-epimerase (RPE1, *YALI0C11880*g) and two dehydrogenases, NADP^+^-dependent glucose-6-phosphate dehydrogenase (ZWF1, *YALI0E22649*g) and NADP^+^-dependent 6-phosphogluconate dehydrogenase (GND1, *YALI0B15598g*), simultaneously with diacylglycerol acyltransferase (DGA1, *YALI0E32769g*) and verified each resulting strain’s ability to synthesize fatty acid growing on both glycerol and glucose as a carbon source. Our results showed that co-expression of DGA1 and TKL1 results in higher SCO synthesis, increasing lipid content by 40% over the control strain (DGA1 overexpression).

**Conclusions:**

Simultaneous overexpression of DGA1 and TKL1 genes results in a higher lipid titer independently from the fermentation conditions, such as carbon source, pH and YE supplementation.

## Background

Nowadays, most global industry is dependent on fossil fuels. Depletion of this source forces scientists to develop alternative substrates for biodiesel feedstock. A suitable replacement for this is biodiesel derived from plants such as sunflowers or canola. Unfortunately, its production requires a huge amount of freshwater and areas of farmlands, which can be applied for the food industry. Thus a decreasing amount of freshwater available for agriculture industry causes that biodiesel derived from vegetable oils becomes ethically doubtful. For this reason a single cell oil (SCO) derived from microbial biomass is a promising replacement for biodiesel in the coming decades. One of the suitable producers of biodiesel precursors is *Yarrowia lipolytica*, an unconventional yeast that is able to produce lipids over 30% of its dry biomass [1, 2] from untypical carbon sources such as alkanes, glycerol or agricultural wastes [3–5]. This well-studied yeast possesses a fully sequenced genome and well-developed genetic tools, including CRISPR/Cas9 [6–8]. Its metabolic engineering or adaptive laboratory evolution (ALE) allowed for the modification of the fatty acid profile, content and employment of a wide range of substrates [9–12]. Moreover, this yeast is able to grow at low pH and use seawater [13], which is an advantage during the fermentation processes on the industrial scale. Lipogenesis is a natural process that occurs in *Y. lipolytica* during nitrogen starvation. Under these conditions, the cells start to accumulate fatty acids in the lipid bodies. The metabolic pathway of lipid synthesis in *Y. lipolytica* has been well studied. A few key factors have been identified in this process, namely malic enzyme (ME), ATP citrate lyase (ACL), acetyl-CoA carboxylase (ACC), glycerol-3-P-*O*-acyltransferase (SCT1), 1-acylglycerol-P-acyltransferase (SLC1), diacylglycerol (DAG) acyltransferase (DGA1 or DGA2) and phospholipid DAG acyltransferase (LRO1) [2]. In particular, it was shown that simple overexpression of the DGA1 (*YALI0E32769g*) gene results in elevated lipid synthesis by *Y. lipolytica* [14]. Moreover, this process required an increased level of NADPH that is provided by NADP^+^-dependent isocitrate dehydrogenase, the oxidative pentose pathway (oxPPP) or malic enzyme [15].

The aim of this study was to improve lipid synthesis in *Y. lipolytica* by co-expression of the DGA1 (*YALI0E32769g*) gene and the genes involved in PPP, transaldolase (TAL1, YALI0F15587g), transketolase (TKL1, YALI0E06479g), ribulose-phosphate 3-epimerase (RPE1, YALI0C11880g) and two dehydrogenases, ZWF1 (YALI0E22649g) and GND1 (YALI0B15598g). Next, we compared the productivity of the process using two different carbon sources: glycerol and glucose. Finally, the influence of the pH on the engineered strain was tested. The highest lipid synthesis was observed during simultaneous overexpression of DGA1 and transketolase (TKL1) genes.

## Methods

### Microorganisms

Strains used in this study are listed in Table 1. These strains belong to the Department of Biotechnology and Food Microbiology at Wroclaw University of Environmental and Life Sciences, Poland.

**Table 1:** Strains used in this study

Strain	Genotype or plasmid	Source
*E. coli*		
DH5α	F^−^ endA1 glnV44 thi-1 recA1 relA1 gyrA96 deoR nupG Φ80dlacZΔM15 Δ(lacZYA-argF)U169, hsdR17(rK- mK+), λ−	[16]
DH5α	pAD-RPE1, *YALI0C11880g*	This study
DH5α	pAD-TKL1, *YALI0E06479*g	[16]
DH5α	pAD-TAL1, *YALI0F15587g*	[16]
DH5α	pAD-GDN1, *YALI0B15598*g	[16]
DH5α	pAD-ZWF1, *YALI0E22649*g	[16]
DH5α	pAD-DGA1, *YALI0E32769g*	[13]
*Y. lipolytica*		
AJD	*MATA*, AJD: ura3-302	[18]
AJD pAD-DGA1	*MATA*, AJD ura3-302, overexpression *YALI0E32769g*	[13]
AJD D/TKL1	*MATA*, AJD: ura3-302, overexpression *YALI0E32769g,YALI0E06479*g	This study
AJD D/TAL1	*MATA*, AJD: ura3-302, overexpression *YALI0E32769g, YALI0F15587g*	This study
AJD D/RPE1	*MATA*, AJD: ura3-302, overexpression *YALI0E32769g, YALI0C11880g*	This study
AJD D/GDN1	*MATA*, AJD: ura3-302, overexpression *YALI0E32769g, YALI0B15598*g	This study
AJD D/ZWF1	*MATA*, AJD: ura3-302, overexpression *YALI0E32769g, YALI0E22649*g	This study

### Media and culture conditions

*Medium* LB (BTL, Poland) was used for cultivation of *Escherichia coli* strains. The inoculum of yeast strains was prepared in Rich Yeast Extract Peptone Glucose (YPD) and it contained: 10 g/L yeast extract (Merck, Germany), 10 g/L peptone (Biocorp, Poland) and 20 g/L glucose (Merck, Germany). The medium for the lipid production consisted of: Medium A: YNB (without amino acids and ammonium sulfate, Sigma, Germany), 50 g/L pure glycerol (POCH, Poland) supplemented with(NH_4_)_2_SO_4_, ratio C/N 60, pH 6.0; Medium B: YNB (w/o aa, w/o ammonium sulfate), 50 g/L pure glycerol, supplemented with (NH_4_)_2_SO_4,_ 0.5 g/L YE, ratio C/N 60, pH 3.0, maintained by 50 mM citrate buffer; Medium C: YNB (w/o aa, w/o ammonium sulfate), 50 g/L glucose (Merck, Germany), supplemented with (NH_4_)_2_SO_4_, 0.5 g/L YE, ratio C/N 60, pH 3.0, maintained by 50 mM citrate buffer.

### Analytical methods

10 mL of samples were spun down (10 min; 4 °C; 5500×*g*), then filtered on 0.45-μm pore membranes and washed twice with distilled water. After drying at 105 °C, the biomass was determined gravimetrically. The fatty acids (FAs) from lyophilized biomass were derivatized to fatty acid methyl esters (FAMEs) using the method described before [13]. FAMEs were analyzed by gas chromatography on GC-2010 Plus apparatus (Shimadzu, Japan) with a flame ionization detector (FID) and autoinjector (AOC-20i). The separation of FAMEs was achieved using a 70% cyanopropyl polysilphenylene-siloxane column (TR-FAME, 30 m × 0.32 mm × 0.25 µm). The initial oven temperature was 130 °C held for 1 min, which was then increased to 200 °C at the rate of 5 °C × min^−1^, then increased to 250 °C at a rate of 10 °C × min^−1^ and held for 1 min. Temperature for the injector and detector were 270 °C and 280 °C, respectively. Helium was used as the carrier gas with constant flow 1.52 mL × min^−1^. Volume of injection was 1 µL with a split rate of 1:5. The identification of FAME was evaluated using Supelco 37 Component Fame Mix as a reference standard and for quantification analysis heptanoic acid was used as an internal standard. The total lipid content in dry cell weight was calculated as the sum of all fatty acids.

### Spark microplate reader (TECAN)

The yeast strain was grown in 96-well plates in 200 μL of YNB medium supplemented with 5% glycerol. First, the strains were grown for 24 h in the same medium, then the cultures were spun down, washed with sterile water and inoculated to an initial OD_600_ of 0.1 in each well. Three biological replications were used in this experiments. The strains were grown at 28 °C under constant agitation with a SPARK microplate reader (TECAN). Growth was monitored measuring optical density at λ_600_ every 30 min for 24 h.

### Shake-flask experiments

The inoculum was grown in YPD medium. Three production media for the shake-flask experiment were used in the study. Production Medium A consisted of: YNB and pure 50 g/L glycerol, (NH_4_)_2_SO_4_ to C/N ratio 60, pH 6.0. Production Medium B consisted of: YNB and pure 50 g/L glycerol, (NH4)_2_SO_4_ to C/N ratio 60, pH 3.0, maintained by addition of citrate buffer. Production Medium C consisted of: YNB and pure 50 g/L glucose, (NH_4_)_2_SO_4_ to C/N ratio 60, pH 3.0, maintained by citrate buffer (50 mM). During shake-flask experiments the cultures were grown in 0.3 L flasks containing 0.03 L of medium on a rotary shaker (CERTOMAT IS, Sartorius Stedim Biotech) at 28 °C at 200 rpm for 120 h.

### Construction of the RPE1 overexpressing strain

First, the RPE1 gene (*YALI0C11880g*) was amplified using primers RPE1-SgSI-F (5ʹ-ATCGGCGCGCCATGGTCCAGCCAATCATC-3ʹ) and RPE1-NheI-R (5ʹ-CTAGCTAGC TGCCGCCTGATTAGGCAG-3ʹ). The obtained 780 bp PCR product was digested with SgSI and NheI and cloned into the corresponding site in the pAD vector [16]. The sequenced plasmid was digested with *MssI* enzyme and subsequently transformed into the strain *Y. lipolytica* AJD pAD-DGA1(*ura*-). The integration in the genome was checked by three independent PCRs.

### RNA isolation and transcript quantification

The cultures were grown in 10 mL of YPD medium in a 100 mL flask. Subsequently the strains were spun down for 1 min at 14,000 rpm. The total RNA was isolated using a Total RNA Mini Plus kit (A&A Biotechnology, Poland) according to the manufacturer’s protocol. Each sample was treated with DNase I (Thermo Scientific) as described in the protocol provided by the company. The RNA quantities were checked by a Biochrom WPA Biowave II spectrophotometer (Biochrom Ltd., UK) equipped with a TrayCell (Hellma Analytics, Germany), then the samples were stored at − 80 °C. The cDNA synthesis was conducted using Maxima First Strand cDNA. The qRT-PCR was analyzed using a DyNAmo Flash SYBR Green qPCR Kit (Thermo Scientific) and the Eco Real-Time PCR System (Illumina, USA). Primers qRPE1-F (5ʹ-TCGGCGCACAATCGCGAATG-3ʹ) and qRPE1-R (5ʹ-GGGCC AAACGAAATGTTGGG-3ʹ) resulted in a 97 bp qRT-PCR product. Other primers for RT-PCR were designed as described before [16]. The results were normalized to the actin gene ACT-F/ACT-R and analyzed using the ddCT method. Samples were analyzed in triplicate.

## Results and discussion

### Overexpression of endogenous DGA1 and PPP genes in *Y. lipolytica* during lipogenesis

**Fig. 1:**
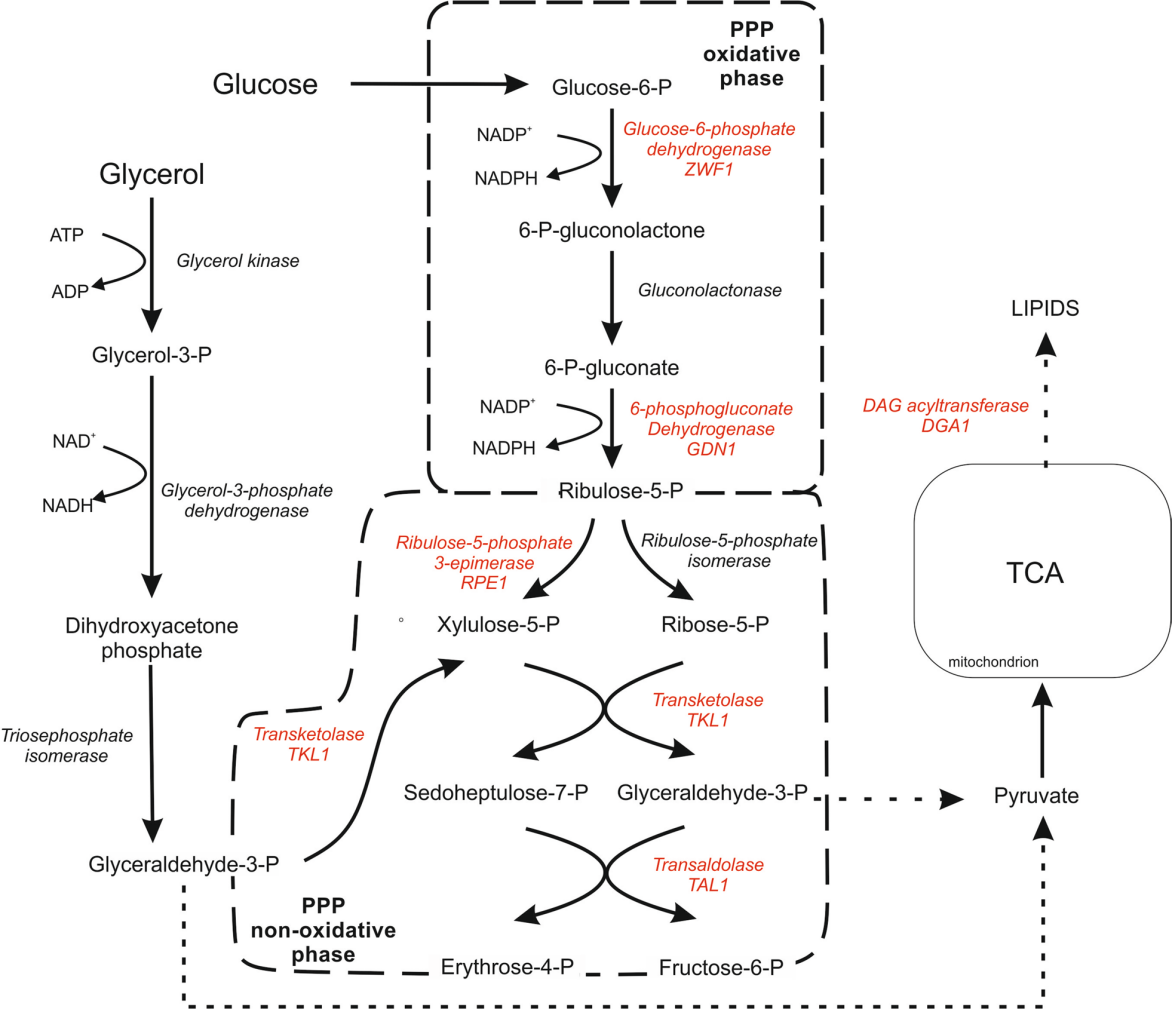
Symbolic pathway of lipid synthesis from glucose or glycerol in yeast *Y. lipolytica*. Dotted lines denotes abridgement of pathway. The gene expressed in this study are labeled in red

In an effort to obtain a high titer of fatty acid we chose a *Y. lipolytica* wild-type strain named A-101 known for high production of biomass [17]. Next, the strain was modified as described before [18]. Because the *DGA1* gene encoding diacylglycerol acyltransferase was found as a key factor of triacylglyceride (TAG) production [19, 20], the vector overexpressing the *DGA1* gene under the UAS1B_16_-TEF promoter was transformed into it, resulting in strain AJD pAD-DGA1 [13]. Biosynthesis of fatty acid and in consequence synthesis of TAG requires huge quantities of NADPH, since fatty acid are a very reduced form. Previously it was shown that during lipogenesis increased demand for NADPH is fulfilled by the pentose phosphate pathway [21]. For this reason, the vectors harboring overexpression cassettes containing genes encoding ribulose-phosphate 3-epimerase (RPE1, YALI0C11880g), transaldolase (*TAL1, YALI0F15587g*), transketolase (*TKL1, YALI0E06479g*), NADP^+^-dependent glucose-6-phosphate dehydrogenase (*ZWF1, YALI0E22649g*) and NADP^+^-dependent 6-phosphogluconate dehydrogenase (*GND1, YALI0B15598g*) were introduced to strain AJD pAD-DGA1, resulting in the strains listed in Table 1. The PPP contains two phases, an oxidative and nonoxidative phase (Fig. 1); thus, at the beginning, glucose 6-phosphate is dehydrogenated, by 6-phosphogluconate dehydrogenase, resulting in NADPH and 6-phosphoglucono-δ-lactone. Consequently, 6-phosphoglucono-δ-lactone is hydrolyzed by a lactonase resulting in 6-phosphogluconate. Next, this compound is oxidatively decarboxylated by 6-phosphogluconate dehydrogenase, giving ribulose-5-phosphate with cogenerating of NADPH. In the second, nonoxidative phase of PPP, transketolase changes xylose-5-P and ribose-P into glyceraldehyde-3-P (GAP) and sedoheptulose-7-P. Subsequently, these compounds are catalyzed by transaldolase, yielding fructose-6-P and erythrose-4-P. Transketolase converts GAP with fructose-6-P into xylulose-5-P and erythrose-4-P. In parallel, GAP is generated in the second stage of glycolysis and subsequently converted into 1,3-bisphosphoglycerate. Consequently, the glycolysis is finished with pyruvate that links with the TCA cycle in the mitochondria. Produced citrate is transported out to the cytoplasm and is converted into oxaloacetate and a molecule of acetyl-CoA [21], which is a substrate for lipid synthesis. This process occurs in endoplasmic reticulum and the produced lipids are stored in the lipid bodies [2].

**Fig. 2:**
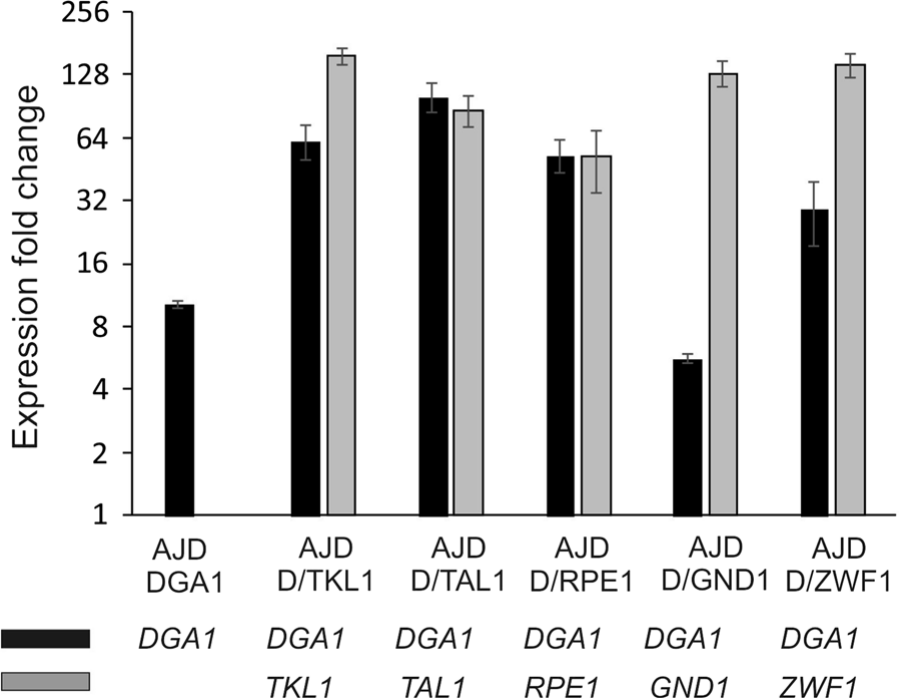
Expression level of genes overexpressed in this study. The reference gene was actin. The samples were prepared in three repetitions. Error bars represent standard deviation

The most common method in metabolic engineering to increase the level of the desired product is functional overexpression of the genes involved in the relevant metabolic pathways. For this reason, the first aim of our study was to evaluate the expression level of the overexpressed genes. For this, we isolated the total RNA from the strains growing for 24 h on YPD medium. As a control the wild-type strain was used. As seen in Fig. 2, all modified strains showed an elevated gene expression level in comparison to the wild type. Surprisingly, despite the fact that that all overexpression cassettes possess the same hybrid UAS1B_16_-TEF promoter, the expression level significantly varies among the strains, but the same effect was observed for this promoter before [15, 20, 22]. The highest activity of this promoter was observed after 24 h of growth [23]. Probably, the strains were not synchronized, and thus the promotor activity varied among them. Despite this fact, here we proved that all genes were overexpressed in the modified strains.

### Growth of the engineered *Y. lipolytica* strains

**Fig. 3:**
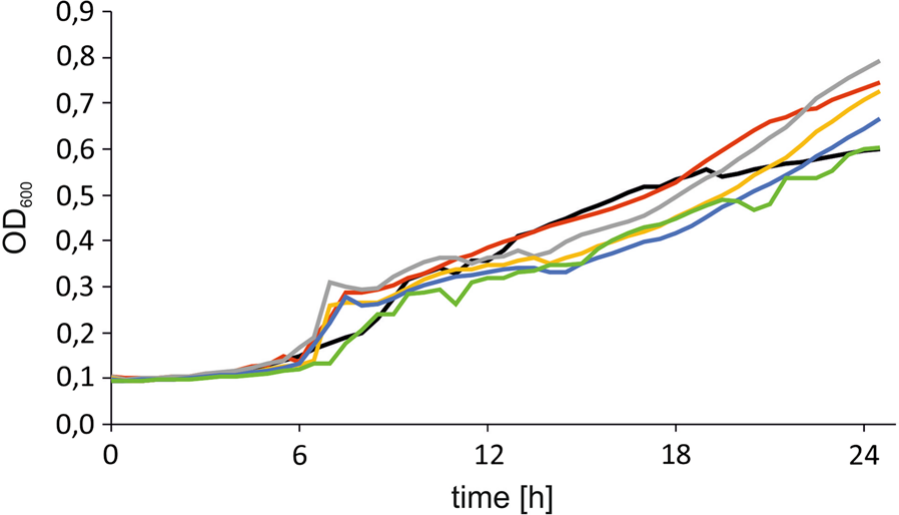
The growth curves of *Y. lipolytica* strains in YNB medium supplemented with glycerol, AJD pAD-DGA1—red, AJD D/TKL1—black, AJD D/TAL1—gray, AJD D/RPE1—yellow, AJD D/GND1—blue, AJD D/ZWF1—green. Growth of yeast strains was measures by increase of optical density (OD_600_) in time in microplate reader Spark Tecan

The metabolic engineering improves the ability of the modified strain to produce the desired metabolites, but sometimes it might impair the functionality of the cell [24]. Therefore, to evaluate whether double overexpression has a negative influence on *Y. lipolytica* growth we performed a growth experiment in a microplate reader. As a carbon source glycerol was used. In this study we employed glycerol as a carbon since it was reported that on this substrate *Y. lipolytica* shows a higher growth rate than on glucose [25] and glycerol is a precursor of triacylglycerols (TAG), the most common form of lipids stored in lipid bodies. As seen in Fig. 3, all the modified strains were able to grow in medium supplemented with glycerol, and no significant delay in growth was observed.

Since the genes overexpression was confirmed, we performed three sets of fermentations for all strains: the first on YNB supplemented with glycerol, pH 6.0 (Medium A), the second on YNB supplemented with glycerol, 0.5 g/L YE, pH 3.0 (Medium B), and the third on YNB supplemented with glucose, 0.5 g/L YE, pH 3.0 (Medium C). In all cases the ratio C/N was 60.

**Fig. 4:**
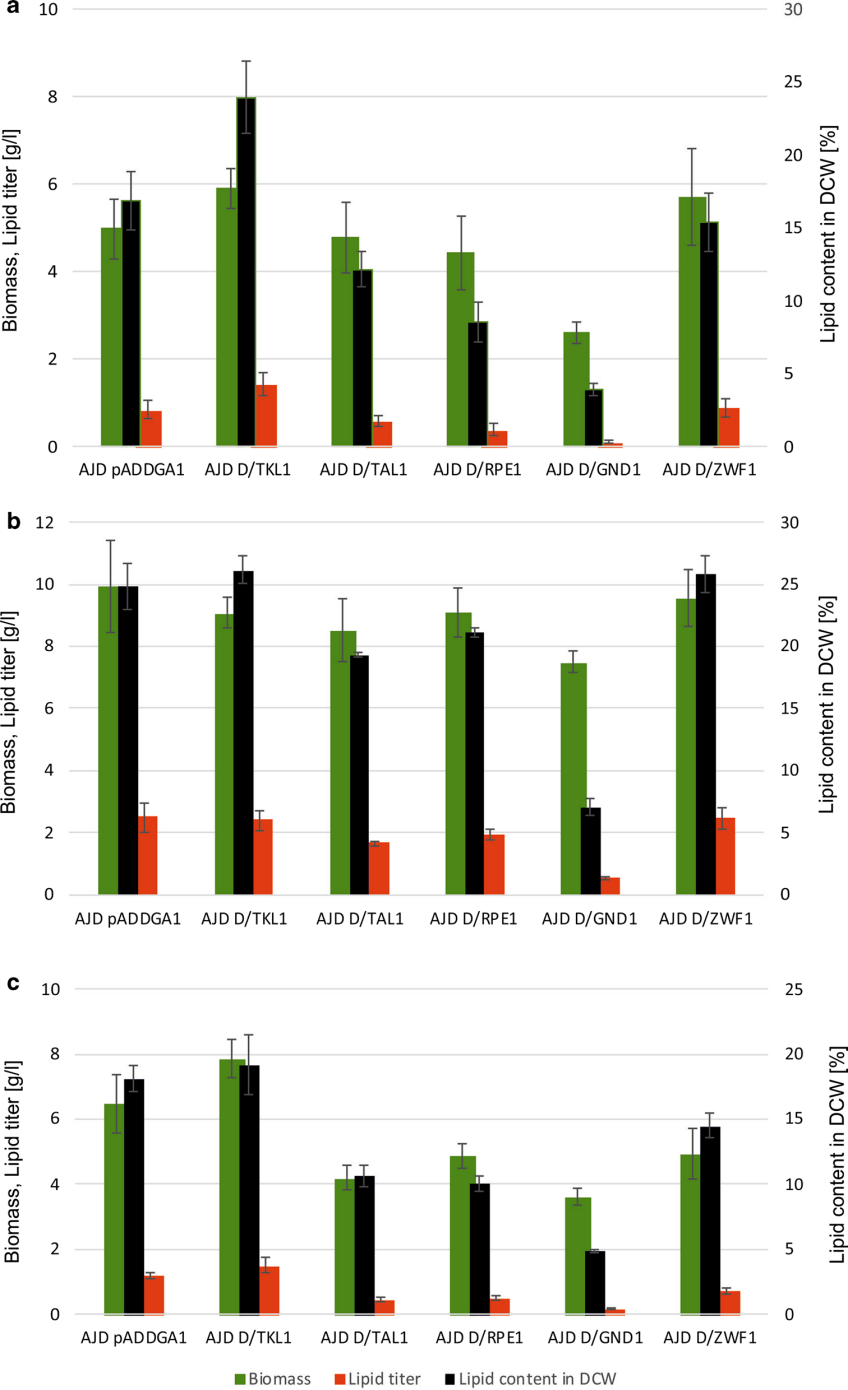
Results of fermentations of the engineered strains in three different media after 120 h. **a** Medium A, YNB supplemented with 50 g/L glycerol, pH 6.0. **b** Medium B, YNB supplemented with 50 g/L glycerol, 0.5 g/L YE, pH 3.0. **c** Medium C, YNB supplemented with 50 g/L glucose, 0.5 g/L YE, pH 3.0. For all media C/N ratio was 60. The obtained values of yeast biomass (g/L)—green bars, lipid titer (g/L)—red bars and lipid content in DCW (%)—black bars are presented. The experiment was prepared in triplicate for each strain. Error bars represent standard deviation

The results of the fermentations are shown in Fig. 4. The biomass production and fatty acid synthesis of each strain were assessed in terms of lipid titer and cellular lipid content. In Medium A (Fig. 4a), the biomass titer was around 5 g/L and the highest biomass was observed for strain AJD D/TKL1 (5.9 g/L). The lowest titer was observed for AJD D/GND1 (2.6 g/L); for the same reason, the lowest lipid titer and content were observed for this strain. The highest lipid synthesis was shown by the strain overexpressing *DGA1* and *TKL1*; the titer was 1.42 g/L and content was 23.94%. This is an improvement by 40% in comparison to the control strain AJD pAD-DGA1 (0.84 g/L, 16.89%, respectively). Overexpression of other genes did not result in a higher biomass titer or in significantly improved lipid synthesis. Co-expression of DGA1 and ZWF1 or GND1 did not result in a higher lipid titer. This result confirms other research where it was shown that simple overexpression of these genes involved in NADPH synthesis did not result in higher lipid production [15]. Surprisingly, we observed lower biomass production and consequently a lower lipid titer in strain AJD D/GND1; however, this strain showed lower DGA1 expression (Fig. 1), and this might explain this result.

Next, we changed the fermentation conditions and the pH was decreased to 3.0 and medium was supplemented with 0.5 g/L YE (Medium B). Under these conditions production of citric acid by *Y. lipolytica* is almost completely inhibited [26, 27], and thus the carbon flux is redirected to biomass synthesis. The obtained results confirm this phenomenon. Under these conditions all tested strains showed significantly higher biomass production and it ranged from 7.5 to 9.93 g/L (Fig. 4b). Therefore, the fatty acid titer was also higher and the content was improved in comparison to Medium A. Again co-expression of *DGA1* and *TKL1* resulted in the highest lipid content, reaching 26.16%. Improvement in fatty acid synthesis was shown by strain AJD D/ZWF1 and the content reached 25.84%. The lowest biomass titer and lipid content were obtained by the strain overexpressing DGA1 and GND1, 7.5 g/L, and 7.09%, respectively. It is worth noting that for the two first strains DGA1 overexpression was maintained at a high level (63- and 30-fold respectively), whereas for the latter it was significantly lower (4.5-fold). This might explain the ineffective lipid accumulation.

Next, to verify whether addition of yeast extract had a significant influence on biomass and lipid production, we performed the shake flask experiment with the engineered strains in Medium C (glucose was used as a main carbon source). Supplementation with YE was maintained at the same level as in Medium B. The results are shown in Fig. 4c. Surprisingly, production of biomass for all strains was lower than in Medium B; thus addition of YE did not have a strong impact on biomass synthesis. The level of biomass titer ranged from 3.60 to 7.87 g/L, for AJD D/GND1 and AJD D/TKL1, respectively. Another strain overexpressing DGA1 and the NADPH generation gene, ZWF1, achieved 4.93 g/L of biomass and 14.51% lipid content. Previously it was shown that overexpression of ZWF1 under the same conditions resulted in similar lipid content, but slightly lower biomass production [15]. Most likely, in our study, *DGA1* expression improved biomass synthesis, but it did not elevate fatty acid content. The strain overexpressing ribulose-phosphate 3-epimerase (RPE1) showed lower titers for both biomass and fatty acid, as under previous conditions. In Medium C the strain overexpressing TKL1 showed the highest titers for both biomass and lipids, 7.87 g/L and 1.50 g/L respectively. The lipid content in this strain was 19.17%. As seen in Fig. 4, supplementation with YE did not have a large impact on biomass synthesis or on lipid titer. The most suitable carbon source for lipid production by *Y. lipolytica* is glycerol. Probably the presence of glycerol in medium simplifies the synthesis of TAG since it is a component of triglyceride. Co-expression of DGA1 and TKL1 genes results in the improvement in fatty acid synthesis independently from fermentation conditions.

### Fatty acid profile in the engineered strain

**Fig. 5:**
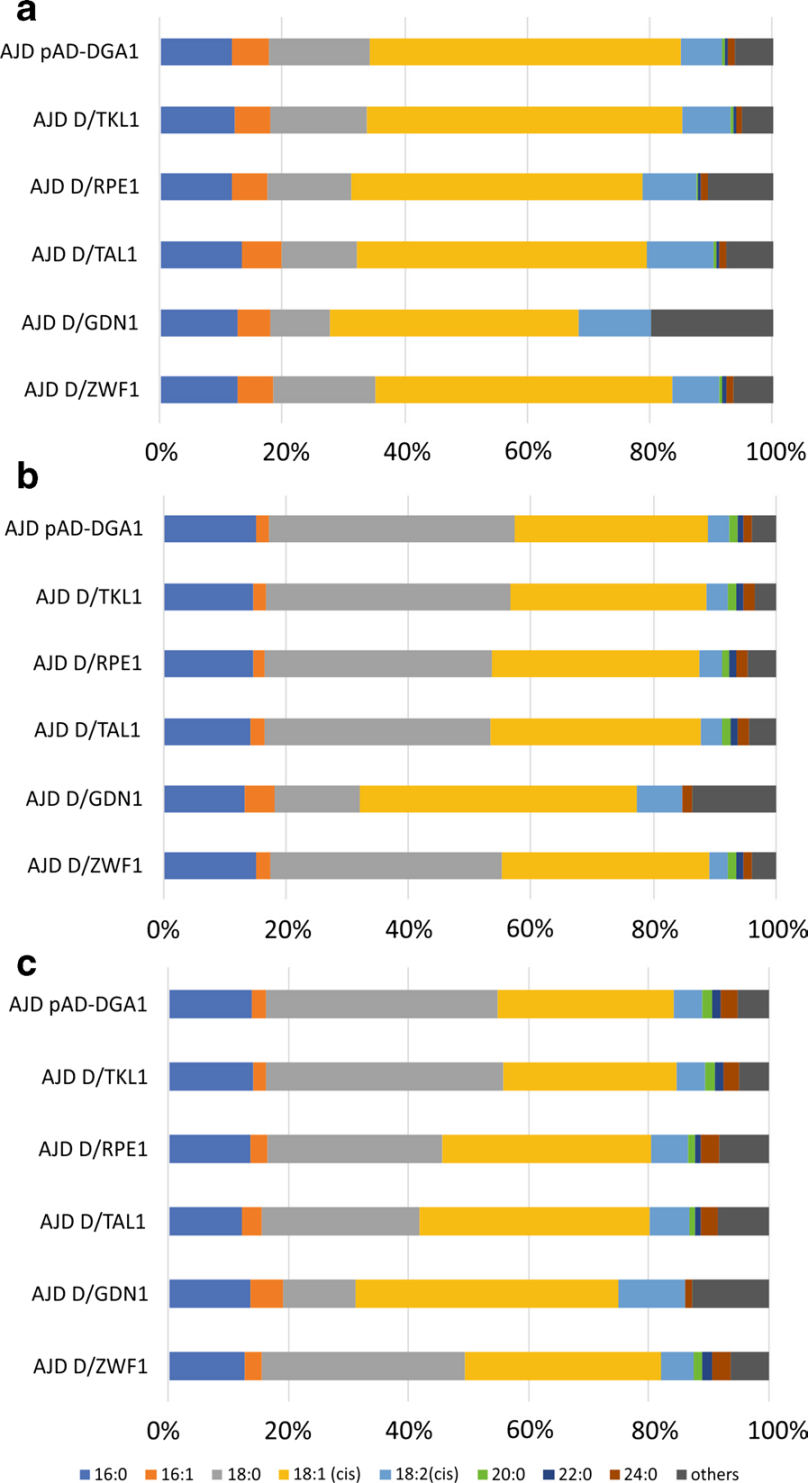
Fatty acid profiles of the engineered strain in three media, Medium A (**a**), Medium B (**b**) and Medium C (**c**). Palmitate (C16:0), palmitoleate (C16:1), stearate (C18:0), oleate (C18:1), linoleate (C18:2), eicosanoic acid (C20:0), docosanoic acid (C22:0), lignoceric acid (C24:0). The bars represents a percentage of fatty acid in tiotal pool of lipid in yeast

Overexpression of gene influences on the total yield and titer of lipid in cells [15, 28] but also fermentation conditions might have an impact on fatty acid composition [5, 29, 30]. In this study in addition to observing the influence on biomass and fatty acid titer, we assessed the influence of PPP genes’ overexpression and conditions on fatty acid profile. The results of that analysis carried out on data from fermentation on three media are shown in Fig. 5.

The obtained data showed that for all media the most abundant fatty acid is oleate (C18:1). In Medium A, its content was around 50% by weight of the total fatty acid level, for all of the strains. The highest concentration was observed for ADJ D/TKL1 (51.5%), the lowest (40.6%) in strain AJD D/GDN1 (Fig. 5a). Overexpression of DGA1 in all tested strains resulted in elevated stearate (C18:0) synthesis; it ranged from 9.7 to 16.5%. This phenomenon has been described before [20], since content of stearate in the fatty acid profile in the wild-type strain grown on glycerol is below 5% [31]. Palmitate was produced at a similar level as stearate and its content was about 12.5% for all strains. Linoleate (C18:2), which belongs to polyunsaturated fatty acids (PUFAs), was produced at a lower level, and its content was below 10% with the exception of AJD D/GDN1 (11.9%) and AJD D/TAL1 (10.8%).

Interestingly, the change of medium pH to 3.0 and addition of YE have an influence on fatty acid profile (Fig. 5b). In Medium B, all strains, except AJD D/GND1, showed significantly higher content of stearate; its pool reached 37–40% by weight of total fatty acid concentration in the engineered strains. Strain AJD D/GND1 accumulated the highest amount of oleate (45.1%), whereas the other strains accumulated below 34%. Overexpression of TKL1 did not have an influence on fatty acid profile; it was almost unchanged in comparison to the control strain (AJD pAD-DGA1). A similar effect was observed in Medium C. Under these conditions, all strains accumulated a higher amount of stearate (Fig. 5c). However, in medium where glucose was the main carbon source, strains AJD D/ZWF1, AJD D/GND1, AJD D/RPE1 and AJD D/TAL produced more oleate (32.5%, 43.5%, 38.3%, 34.9%, respectively) than the control strain (29.3%). Strain AJD D/TKL1 accumulated the highest content of stearate (39.6%), but its profile was the most similar to the control strain. Moreover, apart from the fermentation conditions, co-overexpression of DGA1 and TKL1 showed the highest content of fatty acid in the biomass. Strain AJD D/TKL1 demonstrated the most similar fatty acid profile to the control strain (AJD pAD-DGA1). Although the overexpression of the other genes investigated here showed a rather modest effect on lipid production, their potential role in this process might be still undiscovered. Since lipid synthesis is a complex process, their function might require other factors, and that might be done by metabolic engineering or fermentation optimization.

## Conclusions

The obtained data showed that co-expression of DGA1 and TKL1 in the yeast *Y. lipolytica* allows for robust growth of yeast and improvement in fatty acid synthesis. The strain produced 40% more TAG over the control strain in medium with glycerol as a sole carbon source. Moreover, its fatty acids profile was almost unchanged in comparison to the control strain (DGA1). In addition, these results demonstrate that carbon source and medium supplementation have an influence on fatty acid profile in *Y. lipolytica*.

## Data Availability

The authors promise the availability of supporting data.
